# An Automatized Contextual Marketing System Based on a Wi-Fi Indoor Positioning System

**DOI:** 10.3390/s21103495

**Published:** 2021-05-17

**Authors:** José-Antonio López-Pastor, Antonio-Jesús Ruiz-Ruiz, Antonio-Javier García-Sánchez, José-Luis Gómez-Tornero

**Affiliations:** 1 Neuromobile Marketing Activo Inteligente, SL. Parque Científico de Murcia, Planta 2, Edificio T, Módulo 1, 30100 Murcia, Spain; aruiz@neuromobilemarketing.com; 2 Department of Information and Communication Technologies (TIC), Technical University of Cartagena (UPCT), ETSIT, Campus Muralla del Mar, s/n, 30202 Cartagena, Spain; antoniojavier.garcia@upct.es (A.-J.G.-S.); josel.gomez@upct.es (J.-L.G.-T.)

**Keywords:** indoor location system, smartphone location, area-level positioning, Wi-Fi fingerprint, contextual marketing

## Abstract

A complete contextual marketing platform including an indoor positioning system (IPS) for smartphones is proposed and evaluated to later be deployed in large infrastructures, such as malls. To this end, we design and implement a novel methodology based on location-as-a-service (LAAS), comprising all the required phases of IPS generation: mall digital map creation, the tools/procedures for offline calibration fingerprint acquisition, the location algorithm, the smartphone app acquiring the fingerprint data, and a validation procedure. To select an appropriate fingerprint location algorithm, a comparison among K-nearest neighbors (KNN), support vector machine (SVM), and Freeloc is accomplished by employing a set of different smartphones in two malls and assessing different occupancy levels. We demonstrate that our solution can be quickly deployed at shop level accuracy in any new location, resulting in a robust and scalable proposal.

## 1. Introduction

Nowadays, most large retail companies focus their marketing strategies on knowledge of customer behavior in all the commercial channels. These marketing techniques are known as omni-channel strategies [1]. The overlapping of customers’ online and offline behavior requires awareness [2] to create successful, specific marketing campaigns targeting particular segments of the population. The key is to study both types of behavior before launching any marketing strategy. Online customer behavior is studied in detail through the use of web/app analytics and cookies. Currently, research efforts are mainly addressed at learning about offline behavior; when the customers arrive at points-of-sale. Therefore, aspects such as the time that customers stay inside a given shop, the frequency of these visits, or the routes followed by customers are some of the inputs employed to increase knowledge about potential buyers [3]. Suitable analysis of this information provides marketing departments with the opportunity to optimize efforts which could influence purchasing decisions and define more effective contextual marketing campaigns. However, difficulties arise when these campaigns are carried out in indoor environments in real time. In this case, an indoor positioning system (IPS) is required to learn about consumers’ offline behavior. In order to increase the success of a contextual marketing platform, the system employed to plan and manage an omni-channel marketing campaign should include an IPS module.

Malls are essential locations for retailers. Mall owners often have marketing teams in charge of increasing the frequency of visits, and once they have managed to increase traffic within the mall, they try to influence purchasing decisions to maximize spending. Marketing teams, with a marketing manager at the forefront, are responsible for defining, planning, and managing both online and offline marketing campaigns using contextual marketing platforms. Consequently, malls are obvious environments in which to apply IPSs. As we describe in this work, an IPS is one of the most distinguishing elements of a contextual marketing platform, although we must not forget other technological tools that are necessary to implement a complete and useful solution. The components that must integrate a complete platform to learn about online/offline behaviors are the following: (i) a customer relationship management (CRM); (ii) a content manager system (CMS); (iii) a key performance indicator (KPI) panel; (iv) a mailing and push notification service; (vi) the IPS itself; and (vii) a mobile app. As the first contribution of this work, we delve into the implementation of all the aforementioned elements of a contextual marketing platform, their interconnections, and how their use can increase knowledge about potential customers. The IPS is the module which obtains the location of users inside the mall. Once the position of each visitor is determined, it is an input parameter of the contextual marketing platform. Expressed in a different way, the output of the IPS is employed as input in the contextual marketing platform, providing so-called location-as-a-service functionalities. To this end, customer location and tracking through the use of smartphones inside infrastructures are studied in this work.

As can be observed, the IPS is the engine of the contextual marketing platform. Thus, one of more important aspects is how to accomplish its implementation. In this sense, there is no standard solution that determines position in indoor environments, and this situation provides new engineering challenges. Different alternatives have utilized technologies like Wi-Fi [4], Bluetooth [5], ultrawide band (UWB) [6], or radio frequency identification (RFID) [7]. As Wi-Fi technology is widespread in malls, we discard others, such as Bluetooth, UWB, and RFID technologies. These technologies require the deployment of new infrastructure in the mall with high operational and maintenance costs, increased by the use of beacons and other ad hoc hardware. Moreover, these technologies are out of the coverage range to locate people through a mobile phone.

With respect to the inputs of the location algorithm to implement the IPS module, diverse Wi-Fi smartphone IPS solutions can be found in the scientific literature, such as [8,9], where the so-called inertial measurement unit (IMU) sensors, such as barometers, accelerometers, gyroscopes, or magnetometers, are employed. Merging the data from these sensors with Wi-Fi fingerprint information, precise pedestrian dead reckoning (PDR) can be generated. This system is capable of detection of the direction of movement [8], stop/walk detection, and step counting [9], among other things. The IPS obtained by these sensors along with Wi-Fi fingerprint data can be extremely precise. However, these solutions have a definite disadvantage: they consume a lot of battery power. A system conceived to operate in a large infrastructure during extended time periods must guarantee robust smartphone functionality. Under these premises and prioritizing sustainable battery consumption, one of the design principles of our system is to employ only Wi-Fi received signal strength indicator (RSSI) fingerprint information. Taking this requirement into account, better performance related to battery use and sustainability in comparison with those that employ IMU sensors is ensured, however, location is compromised. In our case of use, a mall, the automatized actions of the contextual marketing platform do not require centimetric accuracy; marketing activities are effective at shop-level granularity. Consequently, we established shop-level precision as a design requirement for our IPS solution.

The drawbacks associated with our proposal are related to the limitations of employing only a Wi-Fi signal: (i) the IPS will be affected by access point (AP) density variations because it depends on the network infrastructure, (ii) smartphone heterogeneity (different manufacturers, models, etc.) implies different RSSI values, even when using the same chipset [10], (iii) the variability of mall occupancy, which impacts aspects such as signal propagation [11], and (iv) the Wi-Fi fingerprint, which frequently changes due to the variation in the number of active APs as time goes by [12]. To the best of our knowledge, there are no studies in the scientific literature comparing Wi-Fi fingerprint algorithms considering these premises in a large, real infrastructure. With the aim of filling this gap, we evaluate three well-known algorithms to be employed as Wi-Fi inputs for our IPS module. These algorithms are the following: K-nearest neighbors (KNN) [13], support vector machines (SVMs) [14], and Freeloc [15]. In a recent study [16], different RSSI fingerprinting indoor location algorithms were compared. The results show that KNN offers appropriate performance through a dataset composed of 4648 fingerprints acquired by using a static procedure during one day in a university building. SVM has been evaluated, showing reasonable accuracy in several indoor scenarios at the shop level [17]. Freeloc can face the problem of dealing with heterogeneous devices and has been extensively evaluated in [18]. Having these inconveniences in mind, as a second contribution, we have conducted an analysis and comparison of IPSs implemented with these three algorithms. Moreover, we have considered the aforementioned problems in malls, such as hardware heterogeneity, variations in AP density over time, and how different occupancy levels affect signal propagation.

The accuracy of the algorithm employed in indoor locations is crucial but the rest of the processes and tools required for the creation of the entire IPS are no less important. In general, the stages deployed to achieve an accurate indoor location solution are defined in [19] and are the following: (i) digital map generation, (ii) the offline calibration phase, (iii) the system build-up/positioning algorithm for localization tasks, and iv) launching and testing. In this work, all the resources and methodology employed in each phase of our IPS, together with every one of the specific processes and tools used to obtain a practical and scalable solution from the perspective of a small–medium enterprise (SME) IT company, are described in detail. Therefore, as our third contribution we have designed, implemented, and thoroughly evaluated a complete IPS module, taking into account the following premises: (i) it employs the already deployed Wi-Fi access points in malls for connectivity purposes, (ii) it considers current market smartphone heterogeneity, (iii) our solution is deployed by non-trained personnel, (iv) it is scalable in all the aspects of the deployment as will be further detailed, and (v) it provides information referring to user location to the contextual marketing platform, resulting in location-as-a-service functionalities.

Concerning the already published works about the implementation of IPSs in large infrastructures, there are studies which provide positioning services in scenarios similar to ours. The COEX mall [19] was the first to be supplied with an indoor location and navigation service based on the analysis of RSSIs. However, several dozens of calibrators along with some weeks of work were required for its implementation. Similar to this work, Li et al. [20] tested their technology in large malls, considering the heterogeneous mobile device problem in these scenarios, offering an accuracy below 2 m. However, ad hoc APs have to be installed in the mall. Mathisen et al. [21] showed a comparative evaluation of several indoor positioning techniques in a large university hospital. In this work, the authors evaluated several IPS solutions from data gathered on different days and at different times, both in static locations and moving throughout the building. The results illustrated the challenges involved in transferring data from a small, controlled environment, such as a small office environment, to a large, dynamic building complex. Furthermore, this work employed samples acquired only on two days and, therefore, did not evaluate the evolution of the different IPSs over the time. Another interesting approach is in [22]. The authors proposed a zone-level IPS by applying neural network techniques, and they compared it against the KNN algorithm, achieving a slight change in accuracy. However, it was deployed in a university building with only 24 APs, and the evolution of the IPS over time was never tested. There are also commercial products for the generation of IPSs employing Wi-Fi signals, like Ekahau RTLS (now renamed to AiristaFlow) [23]. However, these IPSs require an exhaustive calibration and the use of specific and complex calibration tools. Dealing with the drawbacks of the aforementioned IPS solutions is our fourth contribution.

Finally, as a fifth contribution of this paper, we publish a complete dataset fingerprint which provides details of our proposed solution. The database is open to the scientific community including all the information collected during the calibration phase together with the fingerprints acquired in the validation stage. Note that other datasets available for indoor positioning have also been published in the past. In [24], datasets and supporting material employed during the Indoor Positioning and Indoor Navigation (IPIN2017) competition can be found. Another interesting work is by Lohan et al. [16], which provided a full measurement package composed of crowdsourced Wi-Fi fingerprints as well as an intensive review of other indoor Wi-Fi datasets, pointing out their availability, features, and limitations. Mendoza-Silva et al. [25] created a dataset containing measurements collected in a large building for an extended period of time. In this work, the results were obtained by employing a single device. Although this is an important drawback, the time dedicated to this experiment was long enough to detect variations in the Wi-Fi environment and to validate other algorithms. The dataset provided by [26] is also worth mentioning. It includes the following two aspects: (i) RSSI values obtained in an office environment of around 200 m^2^, and (ii) a fingerprint database of magnetic measurements. The main disadvantages of this dataset are the reduced size of the modeled space and the fact that only a single device was used to perform the tests. Some of the authors of this paper also published another dataset [27] which contains RSSI data collected in a large, multi-floor building, by employing several devices carried by different users. This implies collecting geotagged samples which are useful to validate other IPS solutions.

The dataset included in the current paper is available at Zenodo [28], a general-purpose open access repository developed under the European OpenAIRE program. This repository allows researchers to store and publish datasets, software, reports, and any other research-related digital artifacts with the main aim of presenting this information to the rest of the community. Our dataset allows other researchers to carry out their own validation tests since it includes the RSSI measurements collected during the offline phase to build fingerprint models at the shop level. Additional information about our dataset and best practices is provided in the rest of the paper.

To summarize, aside from the extensive comparison and discussion of three of the most well-known algorithms employed in IPS generation (SVM, Freeloc, and KNN), we detail all the considerations necessary to provide a complete and effective contextual marketing platform. The interactions between modules, their architecture, and functionalities are also detailed. This work is structured as follows. Section 2 details the methodology followed for: (i) the architecture of the contextual marketing platform including all the information related to the generation of the IPS, (ii) the malls, smartphones, and validation routes employed in the comparison of the different algorithms, and (iii) the Wi-Fi fingerprints generated and shared in an open access repository. Section 3 illustrates the results of the comparison among the aforementioned algorithms, which are later discussed in Section 4. Finally, Section 5 concludes the paper.

## 2. Methods

This section describes the main modules comprising the contextual marketing platform developed here, focusing on the procedure and tools required for the generation of the IPS. Once the ISP is fully explained, three algorithms (KNN, SVM, and Freeloc) employed to create the engines of IPS solutions, that are key to analyzing its best performance, are detailed. Later, we indicate the malls where the validation procedure was evaluated, the routes selected, and the smartphones employed. Finally, we describe the Wi-Fi fingerprint dataset generated in this work.

### 2.1. Contextual Marketing Platform Architecture

“Marketing automation” is a new paradigm in the relationship between retailers and customers. As was defined in [29], it can be summarized using the following example: “As retailer X, what do we tell customer Y when he/she arrives on Monday morning?”. Marketing automation, together with the omni-channel strategies already mentioned in the Introduction, are faced with the challenge of merging clients’ online and offline behavior. The questions to answer under this paradigm, expressed again through examples, are: “What do we say to a customer who signed up to our mobile app or website two months ago but has never come to our facilities?” and “How do we send the message? Should we send a push notification or should we send an email?”. The answers to these questions are carefully studied by the retailers’ marketing teams and the owner of the mall and implemented as marketing actions. Thus, the automation of these campaigns must be planned since, if not, personnel would have to carry out these actions, and this could make the marketing proposals nonviable. One way to perform effective and automatized marketing campaigns is to use a contextual marketing platform. However, the whole paradigm of contextual marketing, marketing automation, and omni-channel can only be covered if the platform that manages the marketing activities includes an IPS to provide location-as-a-service (LAAS).

In this work, a contextual marketing platform is designed and developed for owners and retailers. It is placed in a mall, and an IPS is implemented to locate and track customers’ smartphones. Explicit permission must be given by users to allow the IPS to know their position inside the mall, the time they spend there, the frequency of their visits, the hour they arrive at the center, etc. These location data are used as input in the automated marketing campaign.

**Figure 1:**
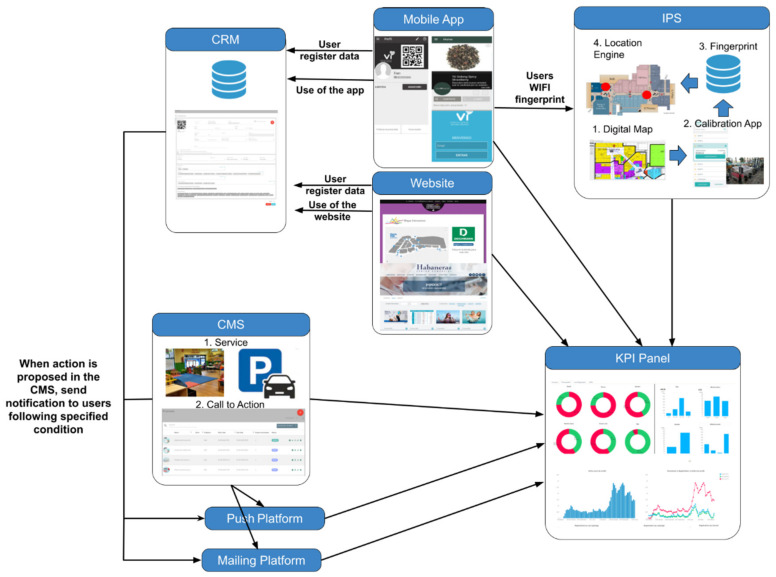
The complete architecture of the contextual marketing platform.

The marketing system stores the information provided by the user in the registration phase both in the app and on the website. Moreover, user interactions with the app and with the website together with their movements in the mall are stored. Meanwhile, mall marketing managers register the campaigns, promotions, and events that could be offered to visitors in the content manager system (CMS). Each of these elements causes different activation mechanisms to interact with users in particular ways. For instance, a specific marketing campaign with some type of discount could be sent by email to users who frequently visited the mall but who have not come in the past two weeks. Another example is that a push notification with a promotion can be sent to customers located in determined areas of the mall to move them to less-visited areas. Analyzing these examples, we can say that without a powerful indoor location system, it would be complicated to implement marketing actions in accordance with the behavior of offline visitors. Thus, these novel functionalities provide added-value tools to marketing managers. The complete detailed architecture of the designed system, including the customer relationship management (CRM), content manager system (CMS), mobile app, indoor positioning system (IPS), push platform, mailing platform, and key performance indicator (KPI) panel, is depicted in Figure 1.

#### 2.1.1. Customer Relationship Management (CRM)

This module of the system (labeled as CRM in Figure 1) is in charge of managing all the information related to the users who have previously registered using the mall website or app. A complete and detailed record of each user is stored with all the information related to her/him. The data employed for the signing-up process, such as email, birth date, marital status, etc., are added to the information generated by the use of the app, the use of the website, and the location information generated by the IPS (if the user gives explicit permission).

The CRM also generates segments of users [30] according to different criteria. In the marketing field, a segment is an aggregation of users with common features or similar affinities. A marketing manager can specify the conditions required to place a user in a specific segment by using the filters available in the module. When new content is created in the CMS, a notification can be sent to one or several segments. If the segment of the user is not relevant to this kind of content, it is published in the mobile app and on the website but the user is not notified. With the use of segments, specific information can be sent only to users who fulfill certain conditions.

With the combination of segments from the CRM and the IPS (explained later), we are able to automatize messages, such as welcome messages or one-day special offers, when users arrive at the mall. Furthermore, surveys can be sent when users leave the shopping centers, asking them to evaluate their shopping experiences to provide quality control.

#### 2.1.2. Content Management System (CMS)

The CMS is the module where the information regarding the mall is introduced to be published (see Figure 1, CMS module). Drupal, WordPress, or Alfresco [31] are some of the well-known CMSs for managing the content of web platforms. In our case, a complete and novel CMS has been developed since we need specific functionalities related to marketing calls-to-action.

The information in the CMS can be related to new proposals, new services available in the mall, or relevant information that managers wish to communicate to their visitors. When the information is completed, with the help of an easy-to-use editor, it can be published on the website, the mobile app, or both. Moreover, an email or push notification can be sent to users in a specific, previously defined segment. The CMS also allows notification triggers to be configured. Some examples of the use of these functionalities are sending emails with information if users have not accessed the website for a month, or sending push notifications with discounts for, for instance, breweries to the users who have checked cinema timetables in the mobile app and have not visited the mall.

#### 2.1.3. Mailing and Push Notification Platform

Nowadays, one of the most effective communication channels in marketing is email. Email campaigns, or email actions, have great impact as they are direct ways of communicating with customers. One of the advantages of our CRM is that it facilitates marketing campaigns using email communications where marketing managers make use of previously defined customer segments. In our proposed solution, information about users’ visits to shopping centers can be employed to establish this segmentation. Considering location data as well as other customer purchasing information, certain patterns of behavior can be estimated, which helps make these email campaigns much more effective. The mailing and push platforms are depicted in Figure 1 below the CMS module.

In the retail context, it is crucial to define the exact moment at which communication with the customer should be carried out; the goal is to contact clients when they are most receptive to the information they are going to receive. Additionally, mailing reception should not provoke customer rejection, resulting in them unsubscribing from the newsletter or uninstalling the app.

The fact that customers install one of our apps in their smartphones implies location services running in the background. This allows us to communicate with them by sending geo-located push notifications. These notifications can be configured in two ways. Firstly, they can be scheduled to be sent at a certain time to a specific customer segment, as discussed above in the case of emails. Secondly, what gives more relevance to this module is knowing where customers are moving and that they receive a geo-located push notification with information about what is happening where they are moving in real time.

#### 2.1.4. KPI Panel

**Figure 2:**
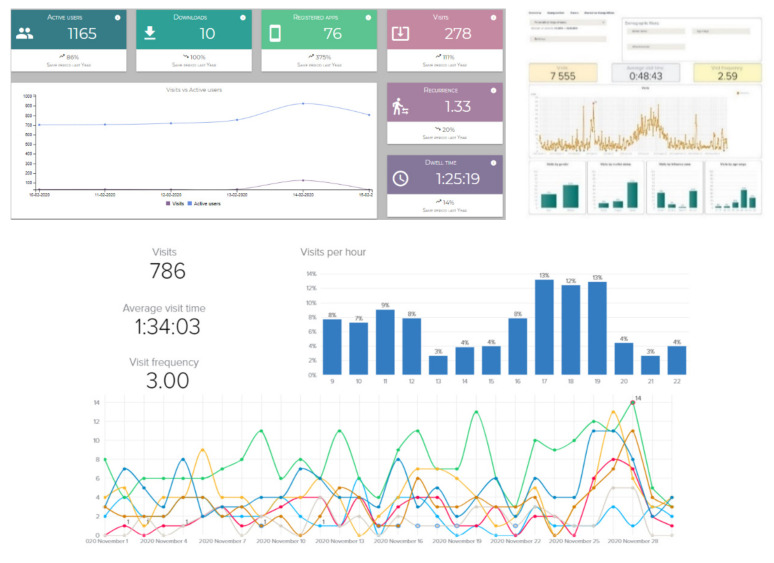
Examples of the KPI panel for marketing purposes in a mall.

One of the most important aspects when evaluating the business model of a mall is to be able to measure the impact of marketing actions. For instance, the possibility of studying the evolution of customers during a Halloween campaign with respect to the data obtained for the same time period of the previous year could be very valuable. The increase or decrease in the number of visits or greater or lesser interaction with the app are issues that can be measured, providing added-value information to determine whether the actions carried out achieve the expected results or not. In other words, our platform, supported mainly by our IPS, becomes a decision-making tool at the management level. Putting this functionality into context with the previous sections, our platform is a complete business-level tool which handles aspects such as operations, communications, special offers, or services, among other things. Figure 2 illustrates the visualization of some KPIs (see Figure 1, KPI module) related to occupancy levels.

#### 2.1.5. Indoor Positioning System (IPS)

As previously mentioned, the IPS (see Figure 1, IPS module) is one of the main elements of the contextual marketing platform. Without the location of the user, the LAAS proposals could not be implemented. This is one of the reasons why our IPS-based solution has been designed with the aim of minimizing the effort needed from personnel in all the deployment phases. This is a real and scalable system that can become the basis of business strategies. Our solution is scalable because it is deployed and maintained with minimum effort in any infrastructure, independently of the country or region where it is located. With these conditions of minimum effort in deployment and maintenance, the system can be used worldwide without an exponential increase in the resources required.

**Figure 3:**
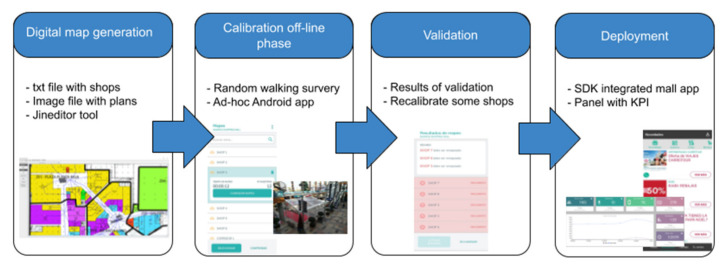
Phases of IPS generation.

Consequently, to achieve a scalable system that can be easily deployed in hundreds of buildings, all the deployment phases must be efficient. The design and implementation of an IPS solution must comprise the phases described in [19] and illustrated in Figure 3, namely: (i) digital map generation, (ii) the offline calibration phase, (iii) a system build-up/location algorithm for localization tasks, and (iv) launching and testing. A fully scalable system can be achieved if the aforementioned phases are performed with reasonable time and effort. For instance, if several workers are required to perform the offline calibration phase over several days, the system is not scalable because it cannot be easily deployed worldwide. Another example is if the computing requirements of the location algorithm and/or the cost of processing the location queries demand high processing capabilities, then the solution is not scalable because locating thousands of visitors is not an affordable task.

In the following subsections, we describe all the considerations referring to the design and implementation of an IPS-based solution in each of its phases. The goal is to demonstrate the feasibility of installing our solution anywhere around the world and providing LAAS to the contextual marketing system.

##### Offline Calibration Phase

According to [19], the acquisition of the radio map (fingerprint of each area to later be used in the location algorithm) during the offline calibration phase is one of the tasks that consumes the most time during the setup procedure of an IPS. In [14], a 3 × 3 m grid was defined with reference points, and fifteen people were employed to calibrate the 10,000 points over two weeks. In our case, this approach cannot be employed because it is not scalable. In accordance with our requirements, the calibration phase has to be performed in a shorter period of time. At the same time, a stable and representative fingerprinting of each area that will be monitored must be acquired over time. Moreover, keeping the aim of scalability in mind, if specific hardware or several well-trained calibrators have to be employed for several weeks, the options for adopting the appropriate solution decrease. To address these drawbacks, we select the *walking random survey method* together with an ad hoc easy-to-use Android app. With this procedure, the person in charge of calibration uses an Android smartphone with our app installed. The calibrator will simply select the area to calibrate in the app, and then he/she will walk around the selected shop/corridor covering most of its surface. The app will collect the RSSI level of the detected APs and will store it together with the label of the area selected. The position and the orientation of the smartphone can randomly change while the calibrator is walking across the shop/corridor. The *walking random survey method* avoids using other time-consuming precalibration tasks, such as reference points.

Once all the areas have been calibrated, the labeled fingerprints are dispatched to the database; they will be the input data in the location algorithm, thus generating the location engine. The offline calibration phase of a mall can be performed in a few hours with the help of a mall staff member employing the calibration Android app and the digital map generator (explained in the next section) together with the calibration procedure.

However, this method of generating a radio map has some disadvantages. First of all, human error is introduced when the calibrator has to select the correct area in the app to label the fingerprint. This can become a source of error because a different location might be selected and the samples would be labeled in the wrong area. This error is easily avoided in malls because the majority of the areas are shops with lighted signs where the name of the display corresponds to the name in the calibration app. Moreover, the app is very user-friendly and intuitive, and the people in charge of calibration can be instructed to avoid this error. Even if an area has been mislabeled, the validation phase, detailed in the following section, has a mechanism to detect this kind of error, and this area can be recalibrated. There is another disadvantage of the *walking random survey* procedure. Although this is a method that allows the fingerprints of a mall to be obtained in a few hours, it has the drawback that changes in the orientation and position of the calibrator can produce drastic changes in RSSI levels. Walking in one direction or another could result in changes of several dB. These changes produce greater errors in comparison with other calibration procedures where IMUs or other smartphone sensors are employed. Nevertheless, the results obtained (and explained in Section 4) illustrate that shop-level accuracy can be achieved with our proposed solution. Therefore, we have chosen a trade-off between an affordable calibration procedure and sufficient accuracy for the system’s purpose.

##### Digital Map Creation

Prior to the calibration phase, a digital map must be created of the relevant mall infrastructure. These areas, along with the calculation of their Euclidean distances, are stored in a database. Then, the calibration app reads the name of the areas to be calibrated from this database. These areas are input parameters to be selected by the person performing the calibration. To generate the digital map, we extended the code of the open-source tool called JIneditor [32], designed by Spatio-Temporal Databases Laboratory at the Pusan National University in South Korea and based on the indoor GML standard [33]. Our system does not require high precision; the shop/corridor level is enough. That is, our solution is far from being a dead reckoning system or an indoor navigation system.

Note that only a list of the shops and the floorplans of the mall are required to generate the digital map. Then, the aforementioned calibration app is installed on any Android smartphone and is fed the names of the shops. A mall staff member will walk with the app turned on, pointing out the shop where she/he is in each instant. Therefore, the samples of the RSSIs of the Wi-Fi APs that are already deployed are stored and labeled with the names of the shops. When all the areas have been covered, the samples are sent to the fingerprint database (see Figure 1) to be employed in the following phases of the IPS creation process.

##### Location Algorithm

The IPS is the module which determines the location of the users inside the infrastructure. Employing the samples acquired from the offline calibration samples, a location engine has to be generated. When a customer with the app installed visits the mall and the RSSIs are sampled, the values are sent to the IPS module, which localizes the position of the user’s smartphone. As previously discussed, due to battery constraints, only the RSSIs of the already deployed Wi-Fi infrastructure is employed. Note that our solution avoids the installation of new hardware infrastructure and the use of IMUs or other additional smartphone sensors.

Several indoor location algorithms for this purpose can be found in the scientific literature [34]. To select which one performs the best, considering our requirements, we have compared three of the best-known indoor location algorithms: k-nearest neighbors (KNN), support vector machines (SVMs), and Freeloc. In a recent comparison of RSSI fingerprinting indoor location algorithms [16], KNN obtains the best result for a dataset acquired in a complete building, as is our case. SVM has been evaluated in several indoor scenarios with proper accuracy at the shop level [17]. Freeloc [15] has also been evaluated and it can have problems with device heterogeneity. However, to the best of our knowledge, there is no study about the performance of the aforementioned algorithms in the case of large infrastructures with shop-level accuracy, hardware heterogeneity, variations in the number of APs over time, and the effects of differing occupancy levels. Details of the different algorithms are presented in the following paragraphs:

- **KNN**. A simple and popular location method which calculates the distances between RSSI fingerprints. When fingerprint scans, including the RSSIs of the APs detected, are acquired, they are sent to a remote server. This server sorts the fingerprints collected in the same order as the ones stored to calculate the distance between them later. Using the Euclidean distance metric, the comparison between the stored fingerprints and the scanned fingerprints follows this equation:
  $$arg\;min_{f_{L}{}_{i}\;\in \;F}\sqrt{\underset{i=1}{\sum }k∥f_{i}-f_{Li}∥}$$
  where $f_{L_{i}}$ is each of the fingerprints acquired during the offline calibration phase in locations $\left\{L_{0},\;L_{1},\;L_{2},\;\ldots ,\;L_{n}\right\}$. Therefore, KNN chooses the area that has the minimum distance between k most similar stored fingerprints.
- **SVM**. A machine learning algorithm which allows discrimination and regression problems to be solved, with the possibility of adapting them to a multi-class situation. The classification is performed by finding the hyperplane that differentiates all the data of one class from the others. In our case, the classes are shops and the data employed for the classification are the RSSIs from the APs acquired in each of the areas.
- **Freeloc**. A heuristic algorithm that generates a unique vector with the sorted APs according to the RSSI values for each location. When a fingerprint has to be classified, it is sorted and compared with each of the stored vectors. The pseudocode of Freeloc is depicted in Algoritthm 1:
  **Algorithm 1** Estimate the location**Input:**$\mathrm{fingerprint}\;\mathrm{data}\;fp_{unknown}$**Output:**estimated location l1:$score\;\leftarrow \;0,\;score_{MAX}\;\leftarrow \;0$2:$value_{MAP}\;\leftarrow \left\{\;\right\}$3:
4:$\mathrm{for}\;\mathrm{each}\;\mathrm{possible}\;fp_{lx}\;\mathrm{in}\;\mathrm{the}\;\mathrm{readio}\;\mathrm{map}\;\mathrm{do}:$5:  $\mathrm{for}\;\mathrm{each}\;KEY_{unknown}\;\mathrm{is}\;fp_{unknown}\;\mathrm{do}:$
6:   $\mathrm{if}\;KEY_{unknown}\;\mathrm{is}\;\mathrm{found}\;\mathrm{in}\;fp_{lx}\;\mathrm{then}:$
7:    $value_{MAP}\;\leftarrow \;VALUE\;\mathrm{vector}\;\mathrm{where}\;\mathrm{its}\;KEY=KEY_{unknown}$
8:    $\mathrm{for}\;\mathrm{each}\;BSSID\;\mathrm{in}\;KEY_{unknown}\;\mathrm{do}:$
9:     $\mathrm{if}\;BSSID\;\mathrm{is}\;\mathrm{found}\;\mathrm{in}\;value_{MAP}\;\mathrm{then}$
10:      score ← score+1
11:     end if12:    end for13:   end if14:  end for15: $\;\mathrm{if}\;score>score_{MAX}\;\mathrm{then}$
16:   $score_{MAX}\;\leftarrow \;score$
17:   
$l\underset{\;}{\leftarrow }l_{x}$
18: end if19:end for20:return *l*

In the Results section, the performance of the different algorithms over time is evaluated by the dataset acquired in two malls using smartphones from different manufacturers.

#### 2.1.6. Location-Based SDK to Overcome IPS Barriers

As described in the Introduction, one of the hardest to solve barriers of IPS solutions and, therefore, our contextual marketing system, is to motivate potential visitors/customers to participate in these localization tasks. To this end, one of the main elements of our scheme is a mobile app. It has to be installed on each visitor’s smartphone to collect fingerprint data while they walk through the different shops. Note that the location task is performed on the server side of the IPS, and the fingerprint data acquired by visitors have to be sent to a remote storage location where the indoor position is computed.

To overcome this barrier, we have developed an easy-to-integrate SDK which can be built into the corporative app of the mall. The mall provides information in its app about movies and other events, special discounts, vouchers, promotions, specific campaigns, etc. Using this tool, along with the mechanism provided by the CRM and CMS, the mall has a direct channel of communication to promote and disseminate specific contents for specific segments of users. When the users install the app, they are invited to receive special promotions once their locations are known. In particular, those users who participate in location data generation through the app can receive advantages in terms of faster Wi-Fi, free Wi-Fi hours, or free playroom services. Once the app has been installed and permission by users has been verified, fingerprint data are obtained every few seconds. This information is compressed, and sent to the server where the IPS is installed. In return, the app notifies the visitors in real time about ad hoc special offers based on their location by means of push notifications.

### 2.2. Validation Scenarios

**Figure 4:**
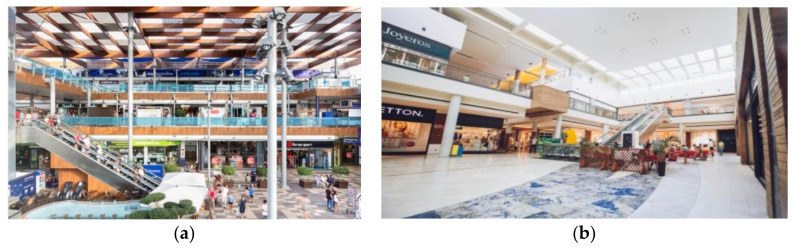
Panoramic image of each of the shopping malls where the IPS has been evaluated: (**a**) mall 1 has three floors and a wooden, cloth-covered roof structure, (**b**) mall 2 has two floors.

Our IPS-based solution has been evaluated in two different single-building malls, both located in the southeast of Spain. Shopping mall 1 is composed of three floors (more than 25,000 square meters) and has a wooden roof structure with big holes covered by a tarp. Mall 2 is divided into two floors and is more than 40,000 square meters. Figure 4 illustrates the different architectural features of mall 1 and mall 2.

Mall 1 has 69 different areas distributed across three floors. Fifty-six of these areas are shops (14 located on the ground floor, 27 on the first floor, and 15 on the second floor). Note that, in our IPS, each shop is a different area. The remaining areas are corridors and a square, with the following distribution: the main square, located on the ground floor, which has been split into two areas; three corridors on the first floor which have been divided into six areas; and, finally, four corridors on the second floor that have been divided into five areas. Therefore, there are 13 areas not categorized as shops. The main reason for splitting the corridors/squares into areas is to avoid large areas since a corridor can traverse the entire shopping center. This is also required to correctly compute the Euclidean distance between areas.

**Figure 5:**
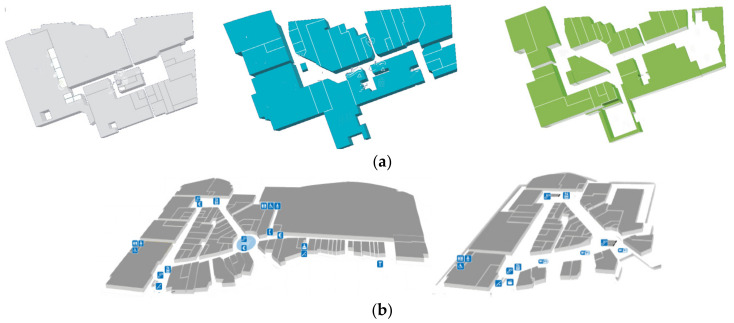
(**a**) Mall 1: ground floor (**left**), first floor (**center**), and second floor (**right**). (**b**) Mall 2: ground floor (**left**) and first floor (**right**).

Mall 2 has 113 areas distributed as follows: 92 shops on two floors corresponding to one area for each shop (59 shops are located on the ground floor and 33 on the first floor); two squares and four corridors located on the ground floor have been split into 12 areas, while two squares and three corridors on the first floor have been divided into nine areas in our IPS. Figure 5 illustrates each floor of mall 1 and mall 2.

### 2.3. Smartphones Employed in the Calibration and Validation Processes

The offline calibration phase and the validation process were performed using different Android smartphones. As is well known, even smartphones sharing the same Wi-Fi chipset measure different RSSI values [10]. The reason for this is that smartphones differ in their antenna orientation and size, in the materials used to make them, or in their software implementation, among other things. It is remarkable that smartphones #9 and #10 are made with the same Wi-Fi chipset; however, #9 is dual-band and supports Wi-Fi standards 802.11 a/b/g/n, while #10 is single-band and only supports standards 802.11 b/g/n. Table 1 details the Android smartphones employed in our study and whose data compose the dataset proposed here.

#### 2.3.1. Offline Calibration Process

Both malls were calibrated by means of the smartphones labeled #1–#5. The main features of these smartphones are shown in Table 1. The time employed to calibrate mall 1 was six hours, while for mall 2, ten hours were required. The calibration process followed the *random walking survey method* described in Section 2.1.5 During the calibration process, five smartphones were carried all at once, without any fixed position/orientation, while the areas were checked. That is, during fingerprint collection, the smartphones were in pockets, handheld, or even in the backpack of the person who was performing the calibration process.

As a rule, the most time-consuming task for deploying an IPS is the offline calibration phase [19]. However, with the proposed *random walking survey method*, this operation can be performed in reasonable time. Moreover, since neither specific smartphones nor well-trained calibrating operators are required, our solution presents greater scalability. The calibration process using the Android app is learned with a few minutes’ training. Additionally, the measurements are taken by employing any Android smartphone. This entails an associated problem: the fingerprints acquired can be very different depending on smartphone characteristics since there are smartphones that implement dual-band technology, have different sampling velocities, are more or less sensitive, etc. Therefore, our location algorithm must be able to derive appropriate location results regardless of the smartphone employed in the calibration phase. In Section 2.4, the differences among the fingerprints acquired with each smartphone are illustrated.

Another remarkable aspect of our calibration process is that normal mall operations were not affected. All the calibrated surface was covered while both malls were open, without considering their occupancy levels and without interfering with their normal routines.

In further sections, we will demonstrate how the performance of our solution is not influenced by occupancy levels (note that the measurement campaign was carried out during opening hours, with varying numbers of clients) and how IPS accuracy does not depend on the smartphone used in the calibration phase.

#### 2.3.2. Validation Routes

Once the calibration phase was finished, four different validation routes were traced in each mall. The objective was to test the accuracy of our IPS-based solution over time but on different days. The routes simulate the movement of visitors/customers during a trip to the mall. Regarding the validation routes in mall 1, 51 of the 69 areas were covered, which was equal to 22,208 square meters out of a total of 25,000. Therefore, 73% of the shops/corridors together with 89% of the total surface of the mall were included in the validation process. Regarding mall 2, 58 of the 113 areas were mapped, covering a surface of 33,4402 square meters (51% of the shops and 83.5% of the total surface of mall 2). The details of each route are illustrated in Table 2. Note that in both malls, the validation routes go through different shops; however, the corridors are shared by different validation routes. Therefore, the total (i) areas and (ii) square meters only compute the different areas.

One of the main purposes of this work is to evaluate the operation of our IPS solution. To this end, we planned four validation routes and several measurement campaigns on different days with different environmental conditions. Moreover, we took into account the variation in the number of visitors in each mall. Table 3 shows the smartphones employed in each of the validation routes, the days elapsed after the offline calibration phase, what route is tested, and the occupancy level. For each validation route, both the position/orientation of each smartphone and the path traced through the shops/corridor are random. That is, the routes follow the predefined shops/corridor, but the movements inside these shops vary from one day to another.

### 2.4. Wi-Fi Fingerprint Dataset Description

The complete dataset obtained from the Android calibration app and described in Section 2.1 employing the aforementioned *random walking survey method* has been uploaded to the Zenodo repository, managed by the European Organization for Nuclear Research (CERN). This repository was developed under the research project named Open Access Infrastructure for Research in Europe (OpenAire) funded by the European Commission. Currently, Zenodo contains a large number of datasets from heterogeneous research fields. The link to access our dataset can be found in [28].

In detail, our dataset is composed of 17,623 fingerprints acquired from five Android smartphones during the offline calibration phase for malls 1 and 2. These fingerprints are divided into 5084 from mall 1 and 12,539 from mall 2. Table 4 shows the different fingerprints acquired by each device, labeling the following aspects: (i) APs detected for each smartphone, (ii) the number of times that the RSSI was acquired, (iii) the number of scans performed, and (iv) APs per scan (on average).

In relation to the validation routes, there were 100,732 validation fingerprints captured from 10 different smartphones; 60,028 validation fingerprints for mall 1 and 40,704 for mall 2. As we worked at the shop level, the total number of fingerprints (calibrating and validation) has been classified into 182 areas, 69 for mall 1 and 113 for mall 2. Concerning the number of APs detected, 885 APs were found in the calibration phase for mall 1 and 1291 for mall 2, whereas 1225 APs were detected in the validation phase for mall 1 and 1752 for mall 2. Compared to other references analyzed in the introduction section [16,24,25,27,45], this complete fingerprint information is one of the largest open access datasets available to the scientific community. In Appendix A, the content of each of the files that make up our dataset is detailed.

## 3. Results

We have evaluated and compared, in terms of stability over time, device heterogeneity, and the influence of occupancy on shop-level accuracy, different IPSs generated following the procedure depicted in Section 2.1.5 by means of the three indoor positioning algorithms: SVM, KNN, and Freeloc. They are described in Section 2.1.5. The evaluation was carried out in two malls, employing the validation routes and smartphones described in Section 2.3. To this end, we have generated five different IPSs with each algorithm using the labeled samples acquired in the offline calibration phase with each one of the #1–#5 smartphones. The validation routes were then processed with each of the IPSs generated, where the inputs are the fingerprint samples to be located, and as output, the placement where they were acquired is estimated. In our case, the output was a shop-level area where the samples were collected.

The performance metric employed to search for the best optimization value is the Euclidean distance from the centroid of the area estimated by the IPS to the centroid of the area labeled in the sample. In the case of a sample for which the target zone is correctly estimated, the Euclidean error is zero. Thus, the shorter the Euclidean distance, the better the performance of the IPS. Moreover, we have employed the cross-validation technique. This means that to verify the performance of the IPS generated from the fingerprints collected by smartphone #1, the validation routes obtained from this smartphone were discarded. This procedure was applied for the remaining smartphones.

The parameters to optimize each of the algorithms are different. In the case of KNN, these parameters are the number of neighbors (APs) employed and the value of the RSSI when an AP is not found in the Wi-Fi scan. In the case of Freeloc, the optimization parameters are the δ and RSS_peak_ [15] employed for the generation of the vector. RSS_peak_ is the RSSI value of the highest frequency during the measurement, while δ is a parameter that must be computed. Finally, in the case of SVM, the parameters are the kernel [17] employed as a classification function and the regularization values (coefficient assigned in the misclassification). For each of the offline fingerprints using smartphones #1–#5 in both malls, we carried out a sweep of the optimization parameters with the aim of finding those with better performance. We tested from 1 to 15 neighbors and 0, 100, and 200 as RSSI values when an AP is not found. In the case of Freeloc, we tested δ from 1 to 10 and RSSpeak from 0 to 16. Regarding SVM, we checked the following kernels: linear, poly, rbf, and sigmoid, with regularizations valued at 0.1, 1, 2, and 3. The values with the lowest errors for the KNN algorithm in both malls and for all the smartphones are three neighbors and 100 for the RSSI when there is no AP. Concerning Freeloc, the optimal values for δ and RSSpeak are shown in Table 5. Regarding the kernel and the regularization parameters for SVM, the optimal values are illustrated in Table 6.

In the rest of the section, we compare the IPSs generated by means of the three indoor positioning algorithms, KNN, Freeloc, and SVM, employing the parameters previously optimized, with the premises of stability over time, device heterogeneity, and the influence of occupancy rates.

### 3.1. Variability of Performance over Time

**Figure 6:**
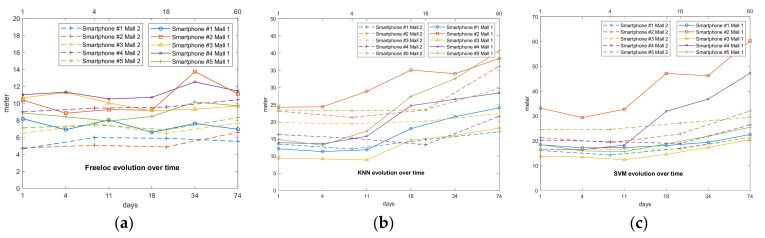
Evolution of the mean error over the time for each of the smartphones in both malls with (**a**) Freeloc, (**b**) KNN, and (**c**) SVM.

To evaluate the three IPSs, the first comparison we conducted was to observe their evolution and performance over time. In works [12,46], they showed great variability with respect to the evolution of the Wi-Fi fingerprint in a large environment. This occurs because APs disappear and others appear. Since offline fingerprint acquisition is one of the most time-consuming tasks and is performed once every several months, the stability of the IPS as time goes by is one of the most important performance indicators. For this purpose, with the samples generated in the offline calibration phase, we generated five different IPSs for each mall with the samples from smartphones #1–#5, to later test the routes labeled #1–#4 over days 1–74 for mall 1, and days 1–60 for mall 2. As has been previously mentioned, we employed the cross-validation procedure and the Euclidean distance error calculated by the difference between shops estimated by the algorithms and the real labeled shop in the sample. The zero-error value is set when a sample is estimated in the area where it was acquired. The evolution of the IPS over time can be observed in Figure 6a for Freeloc, Figure 6b for KNN, and Figure 6c for SVM.

### 3.2. Heterogeneity Diversity Performance

**Figure 7:**
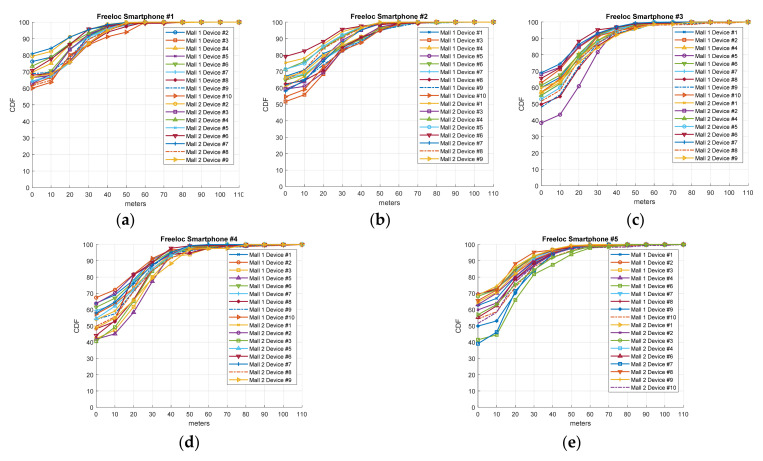
CDF referring to the accuracy of the IPS generated with Freeloc on day 74 for mall #1 and day 60 for mall #2 employing smartphones (**a**) #1, (**b**) #2, (**c**) #3, (**d**) #4, and (**e**) #5.

**Figure 8:**
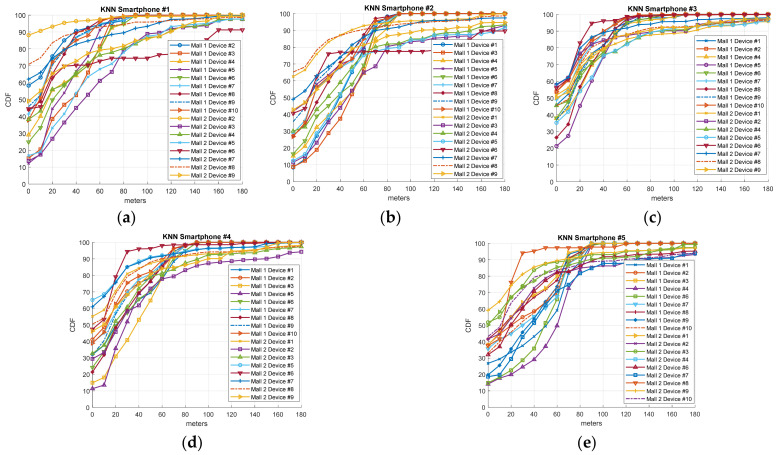
CDF referring to the accuracy of the IPS generated with KNN on day 74 for mall #1 and day 60 for mall #2 employing smartphones (**a**) #1, (**b**) #2, (**c**) #3, (**d**) #4, and (**e**) #5.

**Figure 9:**
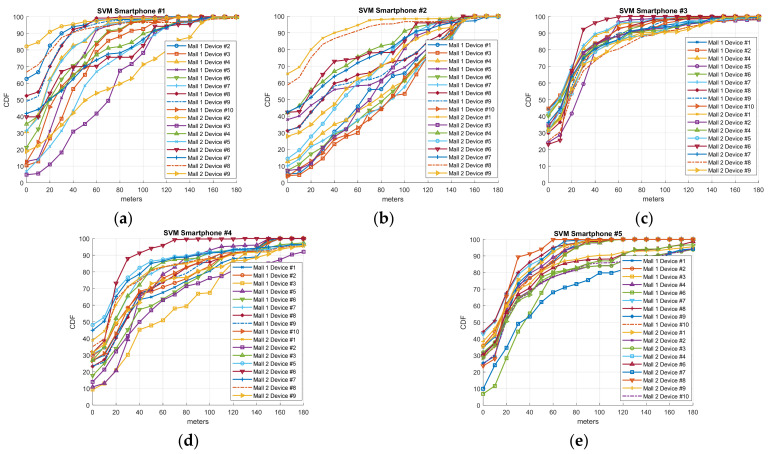
CDF referring to the accuracy of the IPS generated with SVM on day 74 for mall #1 and day 60 for mall #2 employing smartphones (**a**) #1, (**b**) #2, (**c**) #3, (**d**) #4, and (**e**) #5.

As described in [10] and validated in Section 2.4, each smartphone, even those with the same chipset, obtains a very different fingerprint. That is, the data generated by each smartphone in the offline calibration phase differ in RSSI levels, scan rate, and the number of APs detected. Therefore, it is crucial to explore whether the IPS generated by a smartphone from the fingerprint samples gathered has similar accuracy. To this aim, we tested our IPS solution with the routes labeled #1–#4 generated by different devices on day 74 for mall 1 and day 60 for mall 2 after calibration. Note that these days are the last days with evaluation routes. As we have established, ten different smartphones were employed in the routes defined for mall 1 and nine for mall 2. With this process, we can find the indoor location algorithm which has the greatest accuracy regardless of the device employed in the calibration procedure. The results are illustrated in Figure 7 (Freeloc), Figure 8 (KNN), and Figure 9 (SVM) by means of the cumulative distribution function (CDF) metric. CDF depicts the error (measured in meters) of the percentile of samples with a lower error than the indicated one on the x-axis. As in the rest of the Results section, the cross-validation procedure is employed with Euclidean distance as a metric error. In the CDF, the percentage of samples with 0 m errors is the percentage of samples which have successfully estimated the right shop.

### 3.3. Influence of Occupancy Rates

Another factor to be carefully considered is the influence of the fading caused by the number of people in the mall. It is well known that people’s movements affect RSSI levels [11]. In a mall, occupancy ostensibly changes according to the day of the week or even the hour of the day. Typically, there are more people at the weekend or on holidays than on working days. As the calibration phase must not disturb the regular operation of the mall, we cannot control the number of visitors and their frequency. We have studied the performance of Freeloc, KNN, and SVM in terms of mall capacity by considering a single calibration carried out over a few hours.

**Figure 10:**
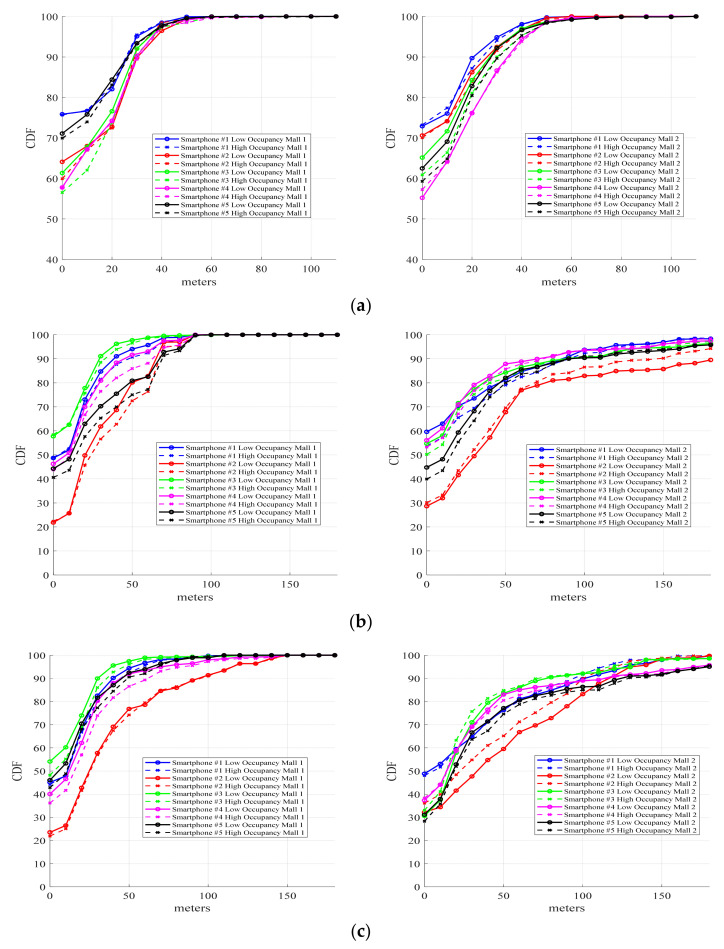
Influence of occupancy rates on the IPS generated with (**a**) Freeloc, (**b**) KNN, and (**c**) SVM.

Therefore, to test the influence of fading on the algorithms, several pre-established routes were followed on the same day but at different times. Specifically, we took routes #2 and #3 for mall 1 on days 11 and 18 after the calibration day, and routes #1 and #4 for mall 2 on day 74. In both malls, the process was completed in the morning when the number of visitors was small, and in the afternoon, when there was a larger number of potential clients. Results employing the CDF metric are illustrated in Figure 10a for Freeloc, Figure 10b for KNN, and Figure 10c for SVM.

### 3.4. Processing Time

Another interesting feature of the IPS involving scalability and the ability to precisely locate a large number of visitors is the processing time of the three different algorithms. To this end, we have evaluated the time employed in determining one of the validation routes generated. The time needed to generate the IPS has been ignored because it is carried out every few months and, therefore, does not affect the overall process. The most critical issue for the appropriate operation of the IPS is to compute the precise location of day-to-day visitors, which can be in the range of a few thousand. Processing time was measured on a PC with an AMD Ryzen 5 and 8 GB of RAM. We selected the route labeled #1, day 1 with smartphone #2, and employed the different IPSs (one per algorithm) generated with smartphone #1. It is a route with 701 fingerprint samples acquired over 1 h and 20 min. The processing time also includes all the routines/code to feed the IPS, like the database extraction of the validation route data, the computation time of the algorithm, and the time spent saving the results in a database. Therefore, the processing time depicted in Table 7 is the amount of time required by the IPS module to compute a regular user visit for each one of the algorithms.

## 4. Discussion

Figure 6 shows the evolution of the performance of the IPS generated by the three algorithms for both malls using smartphones #1–#5. In the case of Freeloc, the results illustrate a flat slope referring to the mean errors (in meters) as time passes, showing an error of around 5 m in the best case (smartphone #2 in mall 2) and 11 m in the worst case (smartphone #4 in mall 1). This means that for most of the IPSs generated using Freeloc, the performance of our IPS on day 74 for mall 1 and on day 60 for mall 2 (that is, the last day of measurement for each mall) is similar to the performance on day 1. In the cases of KNN and SVM, the slope of the mean errors increases as time passes for the entire IPS. Moreover, the mean values of the errors on day 1 for the best performance are, in both cases, around 10 m for smartphone #2 in mall 1. These values are worse than in the case of Freeloc. Therefore, we can conclude that in the case of Freeloc, the error does not increase as time goes by and the system is stable and robust over time. In contrast, in the cases of KNN and SVM, the errors increase over time for the five IPSs generated in both malls. Additionally, it should be noted that in the case of Freeloc, the engines generated for each smartphone in mall 2 present lower errors than the ones generated for mall 1, although mall 2 has a larger surface and a greater number of shops. This is due to the larger number of APs detected in mall 2, which involves a greater number of representative fingerprints for each area.

Regarding the results for device heterogeneity, Figure 7 (Freeloc), Figure 8 (KNN), and Figure 9 (SVM) illustrate the CDF outcomes of the last day of the validation routes for each mall/smartphones #1–#5. It is worth mentioning that errors valued at 0 m on the x-axis are the percentage of samples successfully determined on the right side of the y-axis. In the case of Freeloc, this percentage is around 60% for smartphone #1 and around 40% in the cases of smartphones #3, #4, and #5 (best cases). Concerning KNN, more irregular results are observed than in the case of Freeloc. Therefore, in all the IPSs generated using the five smartphones, the worst performance is around 10% of the samples determined correctly in the right zone. However, 90% of the samples are properly determined in the case of the IPS generated with smartphone #1 for the test device smartphone #2 in mall 2. Similar results can be found for SVM and the five IPSs generated, with values of 0 m errors from 5% to 80% in the case of smartphone #1. Another interesting result to analyze in the case of the CDF is the 80th and 90th percentile. These values illustrate the errors in 80% and 90% of the samples, respectively. In the case of Freeloc, the calculated CDFs show an error of around 30 m in the 80th percentile and 40 m in the 90th percentile, regardless of the smartphone used. It is worth mentioning that misclassification of a sample in our solution entails an error in meters from the centroid of an area to the centroid of another, and the larger the area with errors, the greater the distance of the error. In the case of KNN, the values obtained show a greater error than in the case of Freeloc, with an 80th percentile of around 60 m for smartphone #3 but very poor performance for smartphones #1, #2, #4, and #5. A similar result to that of KNN can be observed with SVM. The results show a similar percentile of error, regardless of the model of smartphone used in the calibration phase, only for the Freeloc algorithm. This demonstrates that any smartphone can perform the calibration process, resulting in a solution that is not dependent on hardware if this algorithm is employed in IPS generation.

With respect to the influence of occupancy levels, Figure 10 illustrates that there are no significant variations in the mean metric error between high and low occupancy once the validation routes have been executed. An exception is smartphone #3, which shows greater error in low occupancy than in high occupancy in samples below the 20 m percentile in both malls; however, for the percentile of distances greater than 30 m (x-axis), a similar error is attained. In the case of KNN, some differences can be found for all the smartphones in both malls and for low/high occupancy. The cases of smartphones #2 and #5 for mall 1 are remarkable as they present better accuracy with low occupancy. In the case of SVM, the most remarkable situation is with smartphone #2 for mall 2, with better results for high rather than low occupancy and without differences in high/low occupancy in other scenarios. Therefore, we can conclude that the IPSs generated by the three algorithms are not affected by occupancy rates.

Finally, the values obtained for the processing time of a route simulating a 1 h 20 min visit to the mall show the worst results in the case of Freeloc, at 11.93 s. In the case of KNN, the processing time is 7.97 s, and for SVM, 8.51 s. As previously mentioned, the computing of the validation route was performed in a non-optimized final user PC, not in a specific computing server. This means that processing times can be drastically reduced. It should be kept in mind that localizing a few thousand users per day is an excellent result in terms of the mean value of numbers of visitors in a large mall. We can conclude that the three algorithms are scalable from a processing time perspective.

In summary, IPS solutions implementing Freeloc have better performance as time passes and in comparison with KNN and SVM, regardless of the type of device used. In terms of occupancy levels, the results show similar accuracy no matter the location algorithm employed. Finally, keeping processing time in mind, the best results are for KNN, although SVM and Freeloc do not show significant differences.

## 5. Conclusions

In this work, a complete contextual marketing platform including the capacity to automatize marketing campaigns, merging online and offline customer behavior, is proposed. The purpose of the system is achieved with a scalable and straightforward Wi-Fi-based indoor positioning system (IPS) at the shop level. Concerning the generation phases of the IPS, one of the most important challenges of the deployment of our IPS-based solution, the fingerprint calibration phase, has been designed and developed using the (i) *random walking survey* method, (ii) an easy-to-use ad hoc Android app, and (iii) a tool based on Jineditor to generate digital maps of the malls. This combination of technologies and procedures could allow us to extend our system to any mall by simply employing the existing APs for connectivity purposes.

Regarding IPS performance, three different indoor positioning algorithms, Freeloc, KNN, and SVM, have been evaluated and compared under the premises of only using Wi-Fi fingerprints as input parameters and without adding new hardware. Regardless of the smartphone employed in the calibration phase, it has been demonstrated that Freeloc has enough accuracy and stability to be employed as a KPI provider in a marketing campaign. Moreover, our solution has been validated under different occupancy scenarios, demonstrating its feasibility as time passes. The performance of our solution remains stable during a substantial time period, with occupancy levels not affecting the results.

The Wi-Fi fingerprint dataset is open access and was generated by means of 10 different smartphones over several weeks in two malls. Furthermore, our dataset provides the RSSI data of movement in shops/corridors of two malls, simulating the movement of visitors during a shopping trip.

Finally, as future work, the implementation of some preprocessing techniques related to calibration fingerprints together with the enforcement of realistic movement models between shops/corridors, such as the hidden Markov model, could improve the performance of the system without increasing computational requirements. Furthermore, thorough studies to discern the preferences of customers split into age groups as well as by their location privacy should be included in future studies.

## Figures and Tables

**Table 1:** Features of the smartphones employed in our study.

Id	Model	Chipset	802.11 Standard	Dual-Band (2.4/5 GHz)	Ref
#1	Xiaomi Mi5	Qualcomm Snapdragon 820	a/b/g/n/ac	Yes	[35]
#2	BQ Aquarius M8	Mediatek MT8167B	b/g/n/ac	Yes	[36]
#3	Huawei P8 Lite 2017	Huawei/HiSilicon HI1101	b/g/n	No	[37]
#4	Huawei P8	Huawei/HiSilicon HI1101	b/g/n	No	[38]
#5	LG K11	Mediatek MT6750	b/g/n	No	[39]
#6	BQ ElCano	Mediatek MT6750	b/g/n	No	[40]
#7	BQ U Lite	Qualcomm Snapdragon 425	b/g/n	No	[41]
#8	Xiaomi Redmi Note 1	Mediatek MTK6592	b/g/n	No	[42]
#9	Xiaomi Redmi Note 4	Qualcomm Snapdragon 625	a/b/g/n	Yes	[43]
#10	Xiaomi Redmi S2	Qualcomm Snapdragon 625	b/g/n	No	[44]

**Table 2:** Routes implemented for validation purposes, showing both the number of areas and the number of shared corridors where RSSI samples were acquired. The square meters covered for each validation route are also indicated.

Route Id	Mall	Number of Areas	Single Shops/Shared Corridors	Square Meters
#1	1	14	9/5	8945
#2	1	19	11/8	7627
#3	1	21	12/9	5831
#4	1	19	10/9	7598
Total Mall 1		51	41/10	22,208
#1	2	17	10/7	5579
#2	2	17	10/7	11,657
#3	2	19	9/10	9279
#4	2	18	9/9	10,027
Total Mall 2		58	38/20	30,642

**Table 3:** Smartphones employed in each validation route, days elapsed after the calibration phase, and occupancy level.

Days	Mall	Route	Smartphones	Occupancy
1	1	#1–#4	#1–#5	Low
4	1	#1–#4	#1–#5	Low
11	1	#1–#4	#1–#5	Low
11	1	#2–#3	#1–#5	High
18	1	#1–#4	#1–#5	Low
18	1	#2–#3	#1–#5	High
38	1	#1–#4	#1–#5	Low
74	1	#1–#4	#1–#10	Low
1	2	#1–#4	#1–#5	Low
4	2	#1–#4	#1–#5	Low
18	2	#1–#4	#1–#5	Low
60	2	#1–#4	#1–#5	Low
60	2	#1–#4	#1–#9	High

**Table 4:** Offline calibration phase: summary of the fingerprints acquired in both malls using the five different smartphones.

Smartphone	Mall	APs Detected (2.4/5 GHz)	Scan Numbers	Average APs per Scan	RSSIs Acquired
#1	1	409/349	1033	49	50637
#2	1	462/398	2345	67	159290
#3	1	383/0	699	21	15185
#4	1	354/0	258	32	6934
#5	1	240/0	749	31	23816
#1	2	664/442	3600	41	150238
#2	2	746/462	6224	52	328355
#3	2	446/0	838	17	14569
#4	2	518/0	1064	22	24123
#5	2	359/0	813	30	24758

**Table 5:** δ and RSS_peak_ optimal values for both malls and smartphones #1–#5.

Smartphone	Mall	δ	RSS_peak_
#1	1	1	0
#2	1	1	2
#3	1	3	0
#4	1	1	0
#5	1	1	0
#1	2	1	1
#2	2	1	2
#3	2	1	0
#4	2	1	0
#5	2	1	0

**Table 6:** Kernel and regularization parameters which have the lowest Euclidean distance errors for SVM.

Smartphone	Mall	Kernel	Regularization Parameter
#1	1	rbf	3
#2	1	rbf	3
#3	1	sigmoid	3
#4	1	sigmoid	3
#5	1	sigmoid	3
#1	2	rbf	3
#2	2	sigmoid	3
#3	2	rbf	3
#4	2	rbf	3
#5	2	rbf	3

**Table 7:** Processing time for the Freeloc, KNN, and SVM for a validation route.

IPS	Time (s)
Freeloc	11.93
KNN	7.97
SVM	8.51

## Data Availability

The data presented in this study are openly available in https://doi.org/10.5281/zenodo.3698238 (accessed on 3 May 2021).

## Appendix A. Structure of the Files within the Dataset

The dataset is made up of eight “.csv” files that can be downloaded and employed without any specific software. The content of each file is described as follows:

- “building.csv”: two registers with the ID of mall 1 and mall 2.
- “floor.csv”: there is an input for each of the floors of the two malls.
- “zones.csv”: for each zone, the mall and the floor in the mall are indicated.
- “training.csv”: in this file are registered the IDs and the description of the five different calibrations performed (one per smartphone) in each of the malls.
- “training_sample.csv”: each file is an RSSI in dBm recorded during the offline calibration phase. The timestamp, channel, and MAC of the AP are also stored.
- “route.csv”: the IDs and the description of the validation routes are indicated in this file.
- “route_sample.csv”: with a similar structure to training_sample.csv, the RSSIs acquired during the validation routes are stored in this file.
- “euclidea_distance.csv”: with the aim of evaluating the error of the different algorithms, this file records the Euclidean distance from the center of a shop to the others in meters.
